# A standard vector for the chromosomal integration and characterization of BioBrick™ parts in *Escherichia coli*

**DOI:** 10.1186/1754-1611-7-12

**Published:** 2013-05-10

**Authors:** Susanna Zucca, Lorenzo Pasotti, Nicolò Politi, Maria Gabriella Cusella De Angelis, Paolo Magni

**Affiliations:** 1 Dipartimento di Ingegneria Industriale e dell’Informazione, Università degli Studi di Pavia, via Ferrata 3, Pavia, Italy; 2 Centro di Ingegneria Tissutale, Università degli Studi di Pavia, via Ferrata 3, Pavia, Italy

## Abstract

**Background:**

The chromosomal integration of biological parts in the host genome enables the engineering of plasmid-free stable strains with single-copy insertions of the desired gene networks. Although different integrative vectors were proposed, no standard pre-assembled genetic tool is available to carry out this task. Synthetic biology concepts can contribute to the development of standardized and user friendly solutions to easily produce engineered strains and to rapidly characterize the desired genetic parts in single-copy context.

**Results:**

In this work we report the design of a novel integrative vector that allows the genomic integration of biological parts compatible with the RFC10, RFC23 and RFC12 BioBrick™ standards in *Escherichia coli*. It can also be specialized by using BioBrick™ parts to target the desired integration site in the host genome. The usefulness of this vector has been demonstrated by integrating a set of BioBrick™ devices in two different loci of the *E. coli* chromosome and by characterizing their activity in single-copy. Construct stability has also been evaluated and compared with plasmid-borne solutions.

**Conclusions:**

Physical modularity of biological parts has been successfully applied to construct a ready-to-engineer BioBrick™ vector, suitable for a stable chromosomal insertion of standard parts via the desired recombination method, i.e. the bacteriophage integration mechanism or homologous recombination. In contrast with previously proposed solutions, it is a pre-assembled vector containing properly-placed restriction sites for the direct transfer of various formats of BioBrick™ parts. This vector can facilitate the characterization of parts avoiding copy number artefacts and the construction of antibiotic resistance-free engineered microbes, suitable for industrial use.

## Background

**Figure 1:**
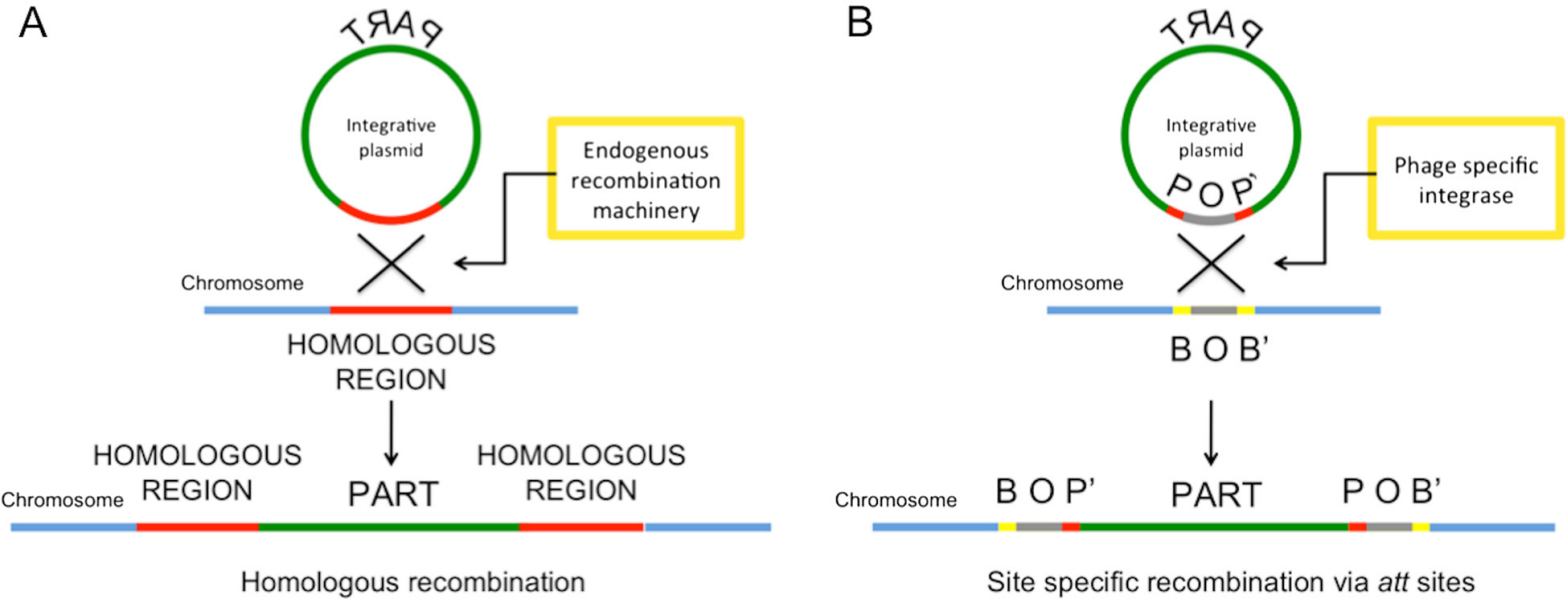
**Main plasmid-based methodologies for chromosomal integration of parts in *E. coli*. A**) Homologous recombination. The integrative plasmid (green) carries a sequence (red) that is homologous with a region (red) in the bacterial chromosome (blue). After a single-crossover event, mediated by the endogenous recombination machinery of *E. coli*, the whole plasmid sequence is integrated in the target region of the chromosome. In specific recA-knockout strains the recombination machinery can be expressed via a helper plasmid. **B**) Site-specific recombination. The integrative plasmid carries a bacteriophage attachment site (attP) that targets the whole plasmid into the specific attachment site (attB) in the host genome. This process is mediated by a specific recombinase that can be expressed via a helper plasmid. The attP and attB sites are composed by the POP’ and BOB’ sequences, respectively. They share a homologous core sequence (O) and different flanking sequences (P, P’, B and B’), so that after site-specific recombination the integrated plasmid is flanked by the BOP’ and POB’ sequences. All the bacteriophage specific *att *sequences have this common structure. In both homologous and site-specific recombinations, positive integrants are usually selected via antibiotic resistance, provided that the integrative plasmid sequence contains an antibiotic resistance marker.

Plasmids are extensively used tools to generate genetically engineered microbes for the expression of recombinant proteins or complex genetic circuits [1-4]. Even if they are very easy to manipulate and incorporate in the desired host, many disadvantages affect their use in both industrial applications and research studies. Common plasmids require the selective pressure of an antibiotic to be maintained in cells, which is costly for industrial scale recombinant protein production [5]. The spreading of antibiotics and resistance markers is also potentially unsafe for the environment [5] and some (e.g., ampicillin) should be avoided in therapeutic protein production because of the potential for human allergic reactions [6]. Selection systems without antibiotics are available, but they require mutant host strains, specific growth media or expensive reagents and in some cases they show low efficiency [5,7]. Plasmids are often replicated in multiple copy in the host cell, which enables the industrial production of a large amount of protein [6]. However, plasmid-free strains with the desired recombinant genes in single copy are required in many studies, e.g., to investigate the effect of these genes in normal physiological conditions, thus avoiding copy-number artefacts [8]. Genome integration can provide the stable insertion of the desired genes in the host chromosome without the need of any antibiotic or resistance marker. Several tools for *Escherichia coli* have been proposed which exploit homologous or site-specific recombination. Homologous recombination can be used to insert the desired DNA fragment (here called *passenger*) into a specific genomic locus that must show sufficient sequence homology with a second DNA fragment (here called *guide*) used to target the locus [9]. This technology can also be used for gene knockout and mutation of native genes [10]. Integrative plasmids (that perform single- or double-crossover) [11], linear PCR fragments [12] and also single-stranded DNA [13] perform the described tasks by means of unspecific endogenous or heterologous protein machinery (e.g., the λRed system [12,13]). On the other hand, site-specific recombination uses genome insertion of bacteriophages in the host chromosome through the phage attachment site (attP) and the bacterial attachment site (attB) sequences [14]. This mechanism has been exploited to develop integrative vectors carrying the attP site (guide) and the passenger [15]. The gene expression machinery that mediates homologous or site-specific recombination can be placed on an easily curable *helper* plasmid transformed in the host strain [9,15]. Figure 1 illustrates how homologous recombination (with a single-crossover event) and site-specific recombination work.

Generally, integrant clones are selected with an antibiotic resistance marker. This marker can be removed by exploiting FRT sites: by flanking a sequence with FRT sites, it can be targeted for excision through the yeast Flp recombinase. Helper plasmids expressing the Flp recombinase have also been constructed [16].

Integrative plasmids must be easily amplified *in vivo*, and clones with a successful integration must be easily selected. To this aim, conditional-replication origins are exploited. They support plasmid replication only in specific conditions, like a specific strain or a temperature range, while the plasmid becomes non-replicative otherwise [10].

For example, the R6K replication origin can be used to propagate integrative plasmids only when the pir or pir-116 gene is present in the host strain [15].

Recent advances in the field of synthetic biology include the standardization of biological parts to facilitate the assembly of genetic circuits [17]. BioBrick™ parts in the Registry of Standard Biological Parts are a rapidly-growing collection of DNA parts that conform to a specific physical standard [18]. BioBrick™-compatible genetic circuits can be easily incorporated in a microbial host through *ad-hoc* constructed BioBrick™ plasmids [19]. Although the construction of integrative systems by using BioBrick™ parts has been reported [20], no standard and ready-to-use solution is available to produce engineered strains with BioBrick™ parts via chromosomal integration. In fact, current tools for site-directed genome integration via integrative vectors include either non-BioBrick™ plasmid vectors with specific, non-customizable DNA guide [15] or BioBrick™-compatible parts [20] that can be used to compose integrative vectors via a standard assembly procedure, but a pre-assembled ready-to-use solution is not available.

In this work, we report the design of a BioBrick™-compatible integrative vector that allows the genomic integration of BioBrick™ parts and can also be specialized to target the desired integration site in the host genome by using BioBrick™ parts. We also demonstrate the usefulness of the designed tool by providing data on the modularity of promoters when characterized in a single-copy context and on plasmids.

## Results and discussion

### Design of the integrative base vector

**Figure 2:**
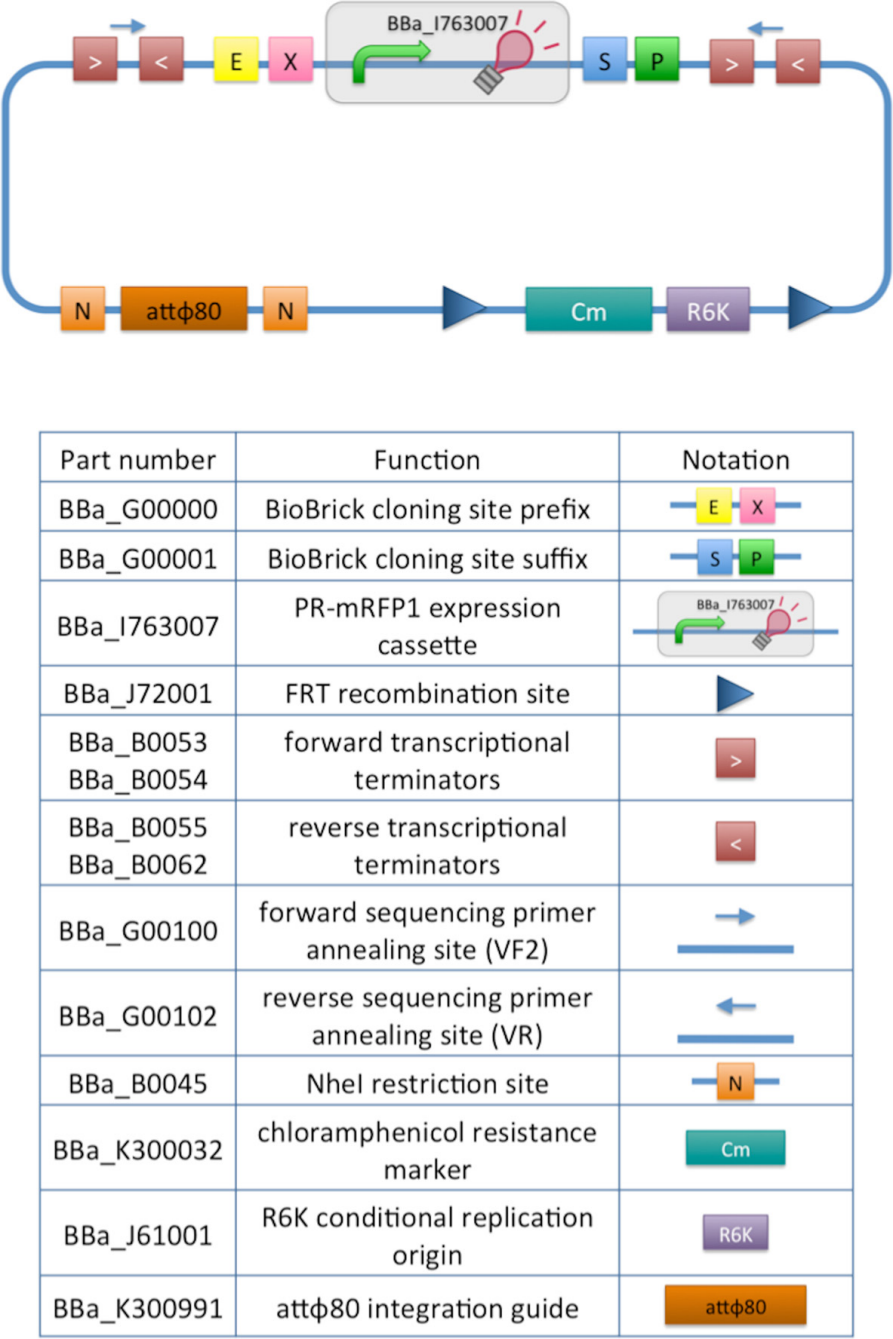
**Integrative base vector pBBintФ structure. **It is a chloramphenicol-resistant vector with a conditional replication origin (R6K) that impairs its replication in commonly used *E. coli *strains (without pir or pir-116 gene). Two FRT sites flank the resistance gene and the conditional origin, so that they can be excised via Flp-mediated recombination once integrated in the genome, thus leaving a marker-less integrant without R6K. This vector targets the Ф80 attB site in the chromosome of *E. coli *via the attP integration guide in the vector. The attP sequence is flanked by two NheI restriction sites to enable the engineering of this base vector by easily changing the guide. The cloning site is flanked by BioBrick™ Prefix and Suffix sequences, while four transcriptional terminators implement the insulation of the part when placed in the genome. Primer binding sites are also present. The default insert of the vector is an mRFP1 constitutive expression cassette, driven by the PR promoter. The glossary explains all the used symbols and lists the BioBrick™ basic parts used to compose this vector.

**Figure 3:**
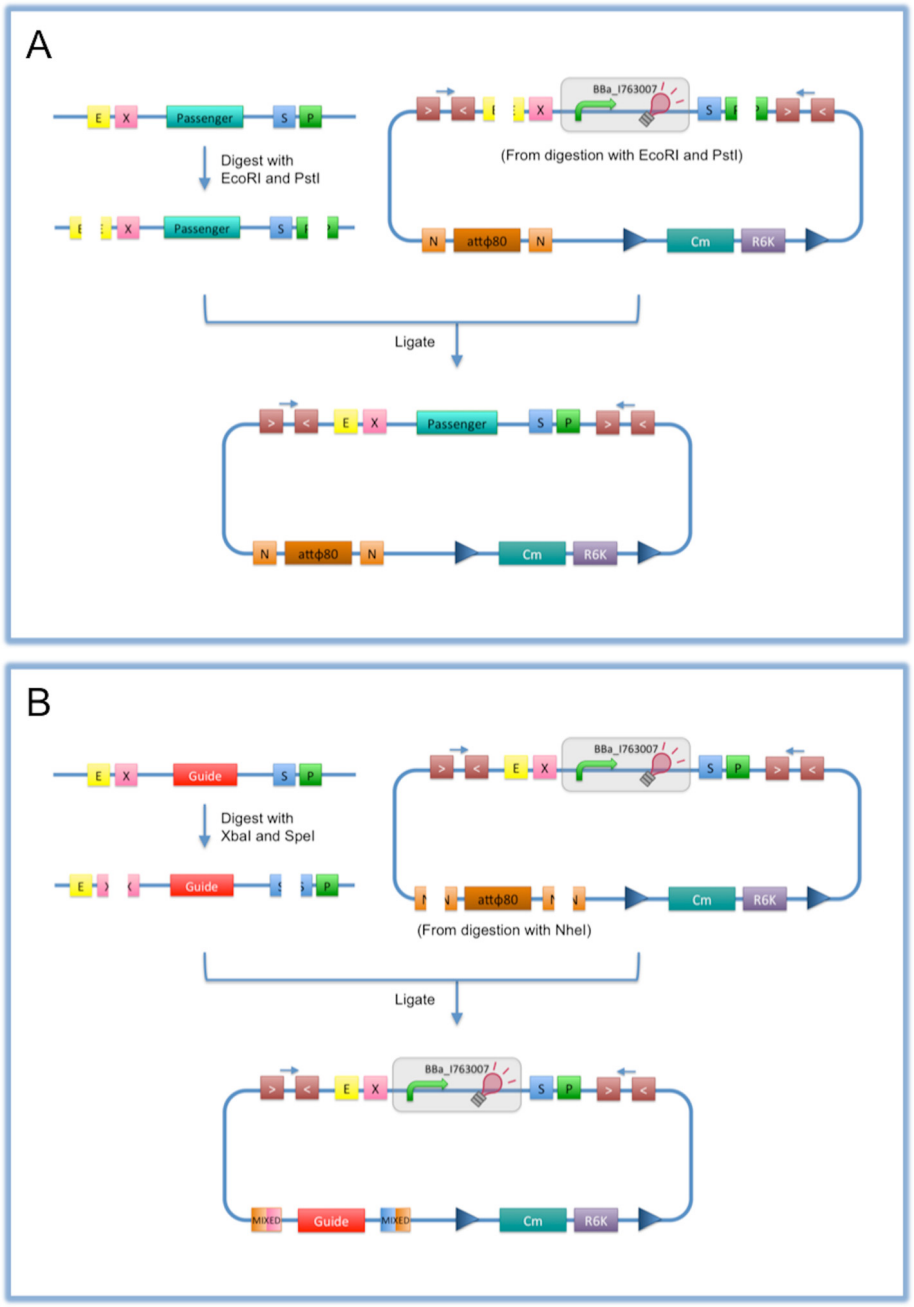
**How to engineer the base vector. A**) The desired BioBrick™ insert can be assembled as passenger in the integrative vector: the BioBrick™ passenger of interest must be digested with EcoRI-PstI, while the integrative vector is also digested with EcoRI-PstI to eliminate the default insert BBa_I763007. The two parts can be ligated and the product has to be transformed into a pir/pir-116 strain to enable the propagation of the vector. **B**) The vector can be specialized with BioBrick™ parts to target the desired locus of the *E. coli *chromosome: the BioBrick™ guide of interest must be digested with XbaI-SpeI, while the integrative vector must be digested with NheI to eliminate the default guide (Ф80 attP). The open vector must be dephosphorylated to avoid self-ligation, the two parts can be ligated and the product has to be transformed into a pir/pir-116 strain. XbaI, SpeI and NheI all have compatible protruding ends. Note that the ligation is not directional, but the guide can work in both directions. The guide can be another attP site or a part which shows a significant homology with a genomic region of the host.

Figure 2 shows the structure of the designed integrative vector, pBBintФ. This base vector uses the default integration guide Ф80 attP, although it can be specialized according to user needs by changing guide and passenger. The cloning site is compatible with the original BioBrick standard (RFC10) and its related standards RFC23 and RFC12, as it is composed by the original BioBrick™ Prefix and Suffix [17]. The presence of illegal restriction sites (XbaI in FRT and SpeI in the Ф80 attP) prevents the usage of this backbone in the classic BioBrick™ Standard Assembly process. However, the presence of unique EcoRI and PstI sites in Prefix and Suffix fully supports the assembly of the desired BioBrick™ parts in the cloning site upon EcoRI-PstI digestion and also supports the 3A Assembly [19,21] (see Figure 3A). The two NheI restriction sites flanking the default integration guide enable the engineering of this backbone by assembling new user-defined BioBrick™ integration guides upon XbaI-SpeI digestion, if the desired guide conforms to the RFC10 or to a compatible standard (see Figure 3B). Although such assembly is non-directional, integration occurs regardless to guide orientation. Like in many other standard vector backbones (e.g., the pSB**5 vector series in the Registry of Standard Biological Parts [18]), the binding sites for standard primers VF2 and VR are present upstream and downstream of the BioBrick™ cloning site respectively. These two sequences are sufficiently distant from the cloning site to enable a good quality sequencing of the insert. The R6K conditional replication origin is necessary to avoid the extra-chromosomal maintenance of the vector during integration; specific strains with the pir or pir-116 genes are required to propagate it during the cloning steps.

The four BioBrick™ transcriptional terminators BBa_B0053, BBa_B0054, BBa_B0055 and BBa_B0062 ensure the transcriptional insulation of the integrated part from its flanking genome sequences, as it is achieved in the pSB**5 vector series. The two FRT recombination sites enable the excision of the R6K origin and the chloramphenicol resistance marker upon Flp recombinase activity. This marker excision allows users to make multiple serial integrations in the same strain in different target loci, always using the same antibiotic resistance marker. The same FRT recombination procedure can also be used, provided that essential chromosomal genes lie between the different target loci (see [12] and [15] for a detailed description of such potential problem when multiple FRT sites are introduced in the same genome).

Additional details about vector sequence, features and construction are available in the BBa_K300000 Registry page.

The engineering of the integration guide allows the integration of parts in user-defined genome positions and for this reason this vector supports the integration by exploiting bacteriophage attP-mediated integration as well as homologous recombination.

In this study, the default insert of pBBintФ is BBa_I763007, a constitutive mRFP1 expression cassette driven by the PR promoter from lambda phage. Note that an early version of the integrative vector included a different default insert that in our experience showed problems, as discussed in Additional file 1: Additional information about integrative base vector design.

### Vector performance validation in the default integration locus

The integration and marker excision capabilities of pBBintФ were tested by integrating a number of BioBrick™ passengers in the chromosome of MG1655 and MC1061 strains. The integrative base vector targets the default integration locus Ф80 attB.

Integration: 100% of the screened colonies (N = 39) on the chloramphenicol plate lost the helper plasmid and 100% of the screened clones (N = 11) showed a correct integration position by PCR (see Additional file 1: Figure S1). 82% of the screened clones (N = 11) had at least two tandem copies of the integrated DNA, as PCR showed (see Additional file 1: Figure S2).

Marker excision: 100% of the screened colonies (N = 100) lost the helper plasmid, while 68% of them had a successful marker excision, validated via chloramphenicol sensitivity. 94% of the chloramphenicol-sensitive screened clones (N = 33) also showed a correct amplicon (see Additional file 1: Figure S3 for a representative experiment) by PCR with primers P1-P4 (see Methods section), thus validating the presence of the construct of desired size in the correct genomic position. In this case, the multiple tandem copies, previously identified by PCR, became a single copy without antibiotic resistance or R6K origin. This tandem copy loss happened because the Flp enzyme excised the entire sequence flanked by the two most distal FRT sites, thus generating a single integrant of the desired construct.

100% of the screened clones with correct P1-P4 amplicon (N = 12) showed the expected sequencing results. 83% of them also showed the expected phenotype. Additional file 1: Additional information about integrated BioBrick™ devices and phenotypes of recombinant strains reports the full list of integrated BioBrick™ devices (Additional file 1: Table S1) and additional information on integration experiments.

### Characterization of a set of BioBrick™ promoters in two different genomic positions

A representative set of widely used BioBrick™ constitutive promoters with the RBS-mRFP1-terminator sequence downstream were integrated in the Ф80 or aspA genomic locus and characterized. The considered BioBrick™ promoters were BBa_J23100, BBa_J23101, BBa_J23118, BBa_I14032 and BBa_R0051, indicated in this work as J23100, J23101, J23118, PlacIQ and PR respectively, while the downstream sequence was the BioBrick™ reporter device BBa_I13507. These constructs were also characterized in the low copy vector pSB4C5, used as a term of comparison. This set of promoters has been previously characterized in different strains and conditions [22].

**Figure 4:**
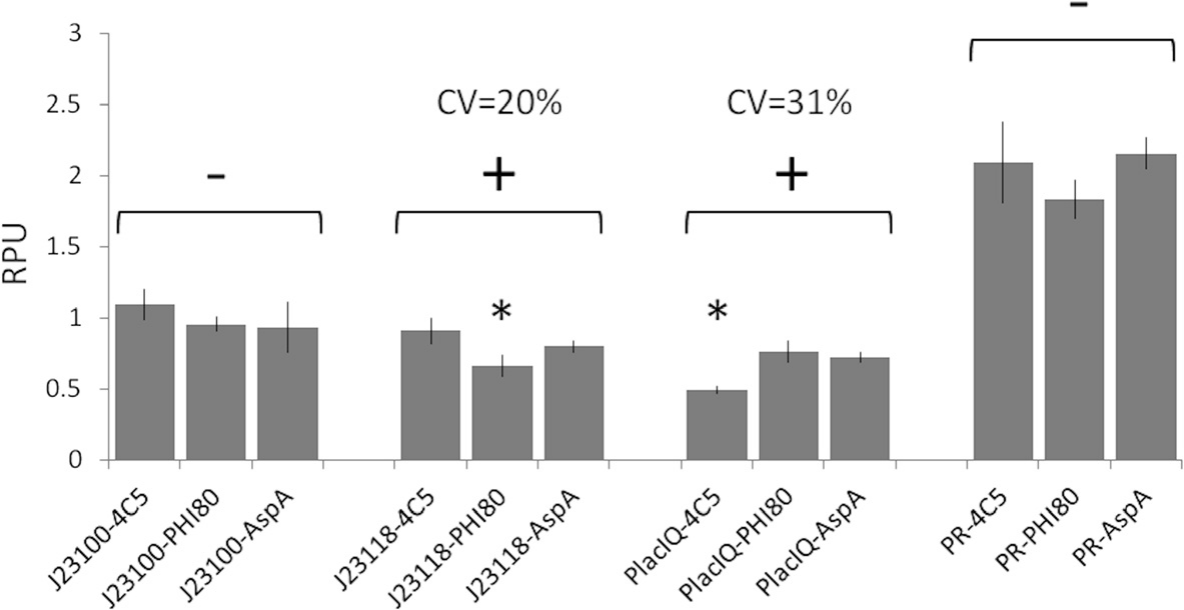
**BioBrick™ promoters characterization, expressed in Relative Promoter Units, in single chromosomal copy (Ф80 and aspA loci) and in low-copy context (pSB4C5 vector). **Promoter activity was measured via the BBa_I13507 (RBS BBa_B0034-mRFP1-double terminator) part. Grey bars represent the average values, computed on at least five clones, and the error bars represent the 95% confidence intervals. For each promoter, statistical analysis was performed via ANOVA test to compare the RPU activities measured in the three different contexts. Conditions showing a statistical difference (P < 0.05) in the mean activities among the three conditions are marked with a ‘+’ sign, while promoters not showing any significant difference (P ≥ 0.05) are marked with a ‘-’ sign. Asterisks represent the post-hoc comparison results. They indicate the individual significantly different condition.

Figure 4 shows the RPU values of promoters measured in the three different physical contexts. Given a promoter and a physical context, the activity of the J23101 promoter in the same context was used as a reference to compute RPUs. While the activity of both J23100 and PR is not statistically different when compared in the three contexts, J23118 and PlacIQ activities show a significant difference in one of the tested contexts with a CV of 20% and 31% respectively. The activity of PlacIQ in plasmid context is different from its activity in the chromosome. This difference could be due to the sequence upstream of the promoter that is identical for the two genomic contexts, but it is different in the pSB4C5 vector which could affect promoter activity [22,23]. The different upstream sequence is not sufficient to explain the difference observed in the J23118 activity, where the promoter shows the same activity in low copy vector and aspA locus, but a slightly lower activity in the Ф80 locus.

**Figure 5:**
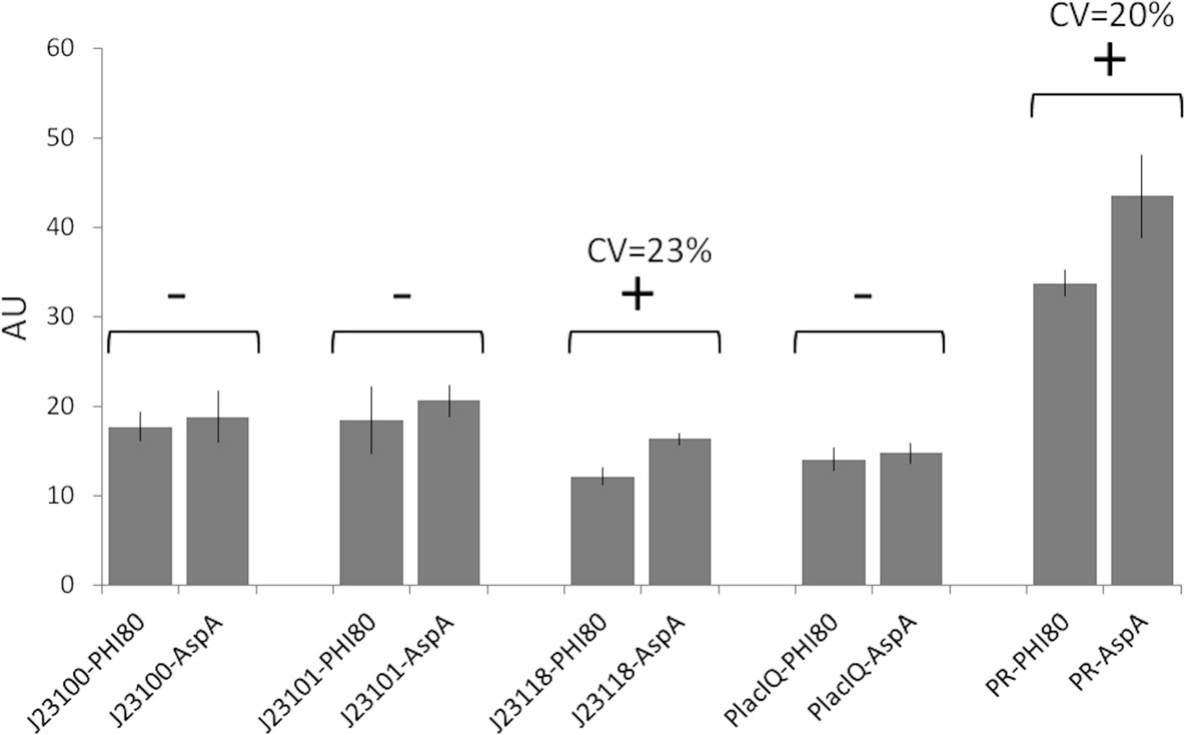
**BioBrick™ promoters characterization, expressed in absolute units (S**_**cell**_**), in the two single-copy contexts (Ф80 and aspA loci). **Promoter activity was measured via the BBa_I13507 (RBS BBa_B0034-mRFP1-double terminator) part. Grey bars represent the average values, computed on at least five clones, and the error bars represent the 95% confidence intervals. For each promoter, statistical analysis was performed via t-test to compare the activities measured in the two different contexts. Comparisons showing a statistical difference (P < 0.05) in the mean activities between the two conditions are marked with a ‘+’ sign, while promoters not showing any significant difference (P ≥ 0.05) are marked with a ‘-’ sign.

Figure 5 shows the absolute promoter activities (S_cell_ value, see Methods section) in the two genomic loci. The five promoters in the aspA locus have a systematically higher activity than in the Ф80 locus, although statistical analysis showed only two promoters with a significant activity difference. These results could be due to several reasons, such as local sequence effects and distance from the replication origin [24]. However, given the complexity of the genome context, the investigation of these effects is beyond the scope of this work.

Finally, the average estimated copy number of the low copy vector is about 7 (measured by dividing the absolute promoter activity in low copy by the one in single copy number), which is consistent with the reported copy number of the pSC101 origin [18,19] and also with previous works [25,26].

### Evolutionary stability of the integrated BioBrick™ devices

**Figure 6:**
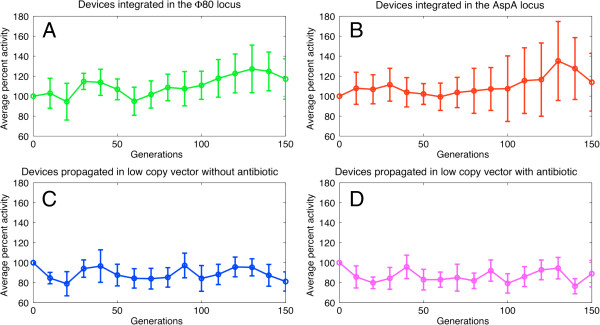
**Evolutionary stability of the studied BioBrick™ promoters expressing RFP.** The devices integrated in the Ф80 (**A**) and aspA (**B**) loci were propagated without antibiotic, while the devices carried on the low-copy vector pSB4C5 were propagated without (**C**) or with (**D**) antibiotic. The four panels show the average percent activity of all the five studied promoters over time in each condition, where 100% represents the average activity at generation 0. For each time point, error bars represent the standard deviations of the measured values.

All the recombinant strains tested in the previous section were also studied during continual bacterial growth for 150 generations. Figure 6 shows the average percent activity, relative to generation 0, of promoters over the generations for each investigated condition: devices integrated in the Ф80 or aspA locus or contained in a low copy vector propagated with or without antibiotic. Additional file 1: Figure S4 shows the results for each individual promoter in the above mentioned conditions. All the recombinant strains are reasonably stable after 150 generations, with only one of the J23100 samples in the aspA locus showing a significant activity loss after >80 generations (see Additional file 1: Figure S4A). Surprisingly, promoter activities are reasonably stable after 150 generations even in the low copy condition without antibiotic. These results show that the majority of the population of the integrated strains likely has not lost the desired function and their stability is comparable with the low copy plasmid condition.

In order to extend the characterization of the propagated cultures, a second experiment was performed on integrated strains and on plasmid-bearing strains grown in presence of antibiotic, at generation 0 and 150. The fluorescence of single clones isolated from glycerol stocks of 150 generation-old cultures was measured to evaluate fluorescence distribution in the population. Single clones from the original glycerol stocks (generation 0) were also assayed for each recombinant strain as a term of comparison. Results are shown in Additional file 1: Figure S5. According to these data and their interquartile ranges (see Additional file 1: Table S2), the variability of integrated strains at generation 150 is not significantly different from the variability of the same cultures at generation 0 (p > 0.05, Wilcoxon signed rank test). The variability of fluorescence in plasmid-bearing strains is lower than in integrated strains for both generation 0 and 150 (p < 0.05, Wilcoxon rank sum test). In accordance with population-based experimental results, a large portion of the clones bearing the J23100 construct (culture 2) in the aspA locus lost their activity. Finally, the averaged fluorescence of the single clones from evolved cultures is highly consistent with the fluorescence of the whole evolved populations, obtained by assaying a culture propagated from glycerol stock (see Additional file 1: Figure S6).

## Conclusions

Several methods have been proposed to target the desired genetic construct into a specific chromosomal locus [27] or even randomly in the genome [28]. Many studies also focused on single-copy integrants generation [15], on the elimination of antibiotic resistance [12,28] or on the copy number amplification of the integrated device [29,30].

Although several genetic tools have been proposed in literature to perform the tasks described above, no plasmid-based standard tools are available to rapidly construct a ready-to-integrate genetic system.

This work describes a BioBrick™ integrative base vector for *E. coli*. Its standard physical interface allows the assembly of the desired parts as passenger or integration guide, both in BioBrick™ format. This vector is desirable to rapidly disclose, via quantitative characterization, if the parts under investigation are suitable in the genomic context and, if required, in single-copy.

The design of this vector was inspired by previous works. In particular, Shetty et al. [19] conceived a BioBrick™ base vector that could be specialized to construct BioBrick™-compatible vector backbones by using BioBrick™ parts as well. For example, vectors with different replication origins or antibiotic resistance markers could be easily assembled from a standard ancestor. Before the work of Shetty et al., the construction of vector backbones required specific genetic manipulations whose knowledge is normally owned by experts. On the other hand, Anderson et al. [20] used BioBrick™ parts to construct different integrative vectors to produce methylase-expressing stable strains without antibiotic resistance marker.

Our work merges such concepts by providing a ready-to-engineer base vector containing all the required features for integration (i.e., a conditional replication origin and an antibiotic resistance marker), marker excision (i.e., FRT sites flanking antibiotic marker and conditional origin), assembly of BioBrick™ parts as passenger (i.e., BioBrick™ Prefix and Suffix) or guide (i.e., NheI sites flanking the default guide). The structure of the vector enables the integration of parts properly isolated from the genomic context. In this way, all the integrated sequences have the same upstream and downstream sequences.

Whatever the integration procedure is, the compatibility of the vector with the target chassis always has to be verified: the presence of the integration locus and, when using site-specific recombination, the absence of prophages in the attB locus must be evaluated according to the declared genotype, genomic databases or by PCR.

While our aim was to provide a physical support to standardize the integrative vector construction that can be used with the desired integration procedure (site specific or homologous recombination by single crossover), this work is not intended to contribute novel integration protocols or methodologies that improve its efficiency. In fact, the efficiency depends on the specific guide, passenger and protocol used. For example, in this work the Ф80 site-specific recombination had a very high success rate while the aspA homologous recombination showed a lower efficiency (see Additional file 1: Additional information about integrative vector efficiency).

A set of BioBrick™ devices, including widely used constitutive promoters assembled with an RFP measurement system, were used as passengers in most of the integration experiments. The behaviour of these devices was quantitatively characterized in single copy in two different loci and in a low copy plasmid. The measured promoter activities were consistent with previous works [22,25]. Given a promoter, its relative activity was comparable among the tested contexts, with a maximum CV of 31% (PlacIQ promoter). Although the different sequence between the upstream regions in the single- and low-copy contexts could be responsible of this activity variation in the case of PlacIQ, the sequence difference could not explain all the observed variability among the contexts. The detected variability entity was consistent with our previous studies on context-dependent variability of these promoters when tested via different measurement systems or when assembled in different genetic circuits [22].

The absolute activity of each integrated promoter was also compared in the two chromosomal positions. Results highlighted a promoter activity difference between the aspA and the Ф80 loci, even if only two of the five promoters showed a statistically significant difference. Although a detailed investigation of such effects is beyond the scope of this work, these results demonstrated the importance of quantitative characterization of parts in different genomic contexts for the predictable design of genetic functions.

Finally, the stability of these integrated BioBrick™ devices has been evaluated via population-based assays and via variability analysis of individual clones. These experiments demonstrated that many integrated strains had comparable stability with plasmid-based ones, even though a small subset of evolved integrated strains showed activity loss. Analysis of the activity of individual clones at generation 0 and 150 showed that variability between the two generations was similar and that integrated strains had higher variability when compared to plasmid-bearing strains. As the used parts were not toxic or hard-to-express parts for the chassis, these stability experiments only represented a proof-of-concept, while the stability of parts has to be tested for each desired construct in the future and it could be dependent on the integration site, DNA sequence, host organism, metabolic burden and/or specific function implemented by the integrated device.

## Methods

### Strains and plasmids

All the *E. coli* strains used in this study are listed in Table 1. MG1655 [31] and MC1061 [32] were used as integration hosts. BW23474 [15] were used to propagate at high copy number the plasmids with the R6K conditional replication origin. TOP10 were heat-shock transformed according to manufacturer’s protocol, while the other strains were made chemically competent as described in [33] and were heat-shock transformed at 42°C for 1 min.

**Table 1:** Strains used in this study

**Strain**	**Relevant genotype**	**Source**
MG1655	wild-type K-12, no Φ80 prophage	CGSC (#7740)
MC1061	no Φ80 prophage	CGSC (#6649)
BW23474	recA1 endA9(del-ins)::FRT uidA4(del)::pir-116	CGSC (#7838)
TOP10	recA1 endA1	Invitrogen

Table 2 lists the plasmids used for the construction of the integrative vectors and the used helper plasmids. The genetic devices used for single copy and low copy characterization of BioBrick™ parts have been reported previously [22,25]. Briefly, all of them are composed by a promoter (BBa_J23100, BBa_J23101, BBa_J23118, BBa_I14032 or BBa_R0051) assembled to the RBS-mRFP1-Terminator sequence (BBa_I13507 reporter device) via BioBrick™ Standard Assembly [17]. For this reason, DNA junctions between the two sequences are identical (i.e. TACTAGAG) for all the constructs. Low copy characterization was performed on the genetic devices in the pSB4C5 BioBrick™ vector.

**Table 2:** Plasmids used for integrative vector construction

**Name**	**BioBrick™ code**	**Description**
pHC-attP-CS	pMK-RQ(BBa_K300983)^a^	DNA fragment including FRT-NheI-attP-NheI-CS(B0033)-FRT, flanked by AvrII sites, in high copy vector
pHC-CmR	pSB1A1(BBa_P1004)^b^	Chloramphenicol resistance gene with promoter in high copy vector
pHC-ter	pSB1AK3(BBa_B0015)^b^	Double terminator in high copy vector
pHC-R6K	pSB1A2(BBa_J61001)^b^	R6K conditional replication origin in high copy vector
pHC-CmRter	pSB1AK3(BBa_K300032)^c^	Chloramphenicol resistance cassette in high copy vector
pHC-PR-RFP	pSB1A2(BBa_I763007)^b^	RFP constitutive expression cassette, driven by the PR promoter in high copy vector
pHC-CmRter-R6K	pSB1A2(BBa_K300008)^c^	Chloramphenicol resistance cassette with R6K origin downstream in high copy vector
pBBintФ-B0033	BBa_K300982^c^	Integrative base vector, targeting the Ф80 locus, with RBS BBa_B0033 as insert
pBBintФ-RFP	BBa_K300000(BBa_I763007)^c^	Integrative base vector, targeting the Ф80 locus, with RFP constitutive expression cassette as insert
pHC-aspA	pSB2K3(BBa_C0083)^b^	aspA coding sequence in high copy vector
pBBintAsp-RFP	BBa_J107058(BBa_I763007)^c^	Integrative vector, specialized to target the aspA locus, with RFP constitutive expression cassette as insert
pInt80-649	BBa_J72008^d^	Ampicillin-resistant low copy helper plasmid, with temperature-sensitive pSC101 origin, containing a constitutively expressed pir-116 gene and the site-specific recombination machinery of the Ф80 phage under the control of a heat-sensitive promoter.
pCP20	not in the Registry^e^	Ampicillin- and chloramphenicol-resistant low copy helper plasmid, with temperature-sensitive pSC101 origin, containing the yeast Flp recombinase under the control of a heat-sensitive promoter.

The BioBrick™ code of the plasmids include the vector name out of brackets and insert name in brackets.

^a^: obtained from Mr. Gene DNA synthesis service. pMK-RQ is a kanamycin-resistant high copy number delivery vector from Mr. Gene.

^b^: obtained from DNA Distributions of the MIT Registry of Standard Biological Parts.

^c^: constructed in this study.

^d^: gift from the Dr. J.C. Anderson lab (UC Berkeley).

^e^: obtained from CGSC (plasmid purified from recombinant strain CGSC#7629).

### Cloning methods

Plasmids were propagated in recombinant strains grown in selective Luria-broth (LB) [33] at 37°C, except plasmids containing a temperature-sensitive replication origin that were grown at 30°C. Antibiotics were routinely used for plasmid maintenance during DNA propagation: ampicillin (100 mg/l for high copy plasmids, 50 mg/l for low copy plasmids), chloramphenicol (12.5 mg/l) and kanamycin (50 mg/l). Liquid cultures (5 ml) were incubated with shaking at 220 rpm. DNA was extracted with the NucleoSpin Plasmid kit (Macherey-Nagel) from recombinant cultures grown to saturation. Purified DNA was digested as required and gel-extracted with the NucleoSpin Extract II kit (Macherey-Nagel). DNA parts were ligated to construct all the plasmids described in this work. 1 μl of ligation mix was transformed into a proper *E. coli* strain, DNA was purified and screened via restriction digest and electrophoresis. Sequencing was performed to validate all the plasmids with primers VF2 (TGCCACCTGACGTCTAAGAA) and VR (ATTACCGCCTTTGAGTGAGC). Long-term bacterial stocks were prepared by mixing 750 μl of a saturated culture with 250 μl of 80% glycerol and stored at -80°C.

NheI restriction enzyme is from Fermentas. Antarctic Phosphatase is from New England Biolabs. Platinum Taq DNA polymerase (Invitrogen) or Phusion Hot Start Flex DNA Polymerase (New England Biolabs) were used to perform PCR. All the other DNA-modifying enzymes are from Roche Diagnostics. Enzymes have been used according to manufacturer’s protocols. Oligonucleotides were purchased from Primm (San Raffaele Biomedical Science Park, Milan, Italy) or from Sigma Aldrich. Sequencing was performed by BMR Genomics (Padua, Italy) DNA analysis service.

### Integrative base vector construction procedure

Additional file 1: Figure S7 shows the assembly scheme followed to construct the pBBintФ vector backbone. Briefly, pHC-CmRter was constructed via BioBrick™ Standard Assembly by assembling the insert of pHC-CmR, digested with EcoRI-SpeI, to pHC-ter digested with EcoRI-XbaI. Analogously, pHC-CmRter-R6K was obtained by assembling the insert of pHC-CmRter, digested with EcoRI-SpeI, to pHC-R6K digested with EcoRI-XbaI. The insert of pHC-attP-CS is composed by, in this order, FRT-NheI-attP-NheI-CS(BBa_B0033)-FRT, where CS is a cloning site composed by EcoRI and PstI with four transcriptional terminators flanking them and two standard primer binding sites for VF2 and VR (see Figure 2). In this plasmid, the CS contains the small BioBrick™ RBS BBa_B0033 between EcoRI and PstI. The insert of pHC-attP-CS was excised through digestion with AvrII and it was assembled with the insert of pHC-CmRter-R6K, which had been excised from its vector through digestion with XbaI-SpeI and dephosphorylated, to obtain pBBintФ-B0033.

Finally, pBBintФ-RFP was obtained by assembling the insert of pHC-PR-RFP, digested with EcoRI-PstI, to the vector backbone of pBBintФ-B0033, digested with EcoRI-PstI in order to cut out and eliminate the BBa_B0033 RBS.

### Assembly of the desired passenger and guide

The source or assembly procedure of all the BioBrick™ constructs used as passengers has been reported previously [22,25]. These constructs have been excised from their original vector backbone upon EcoRI-PstI double digest and they have been cloned into the pBBintФ or pBBintAsp vector as described in Figure 3A.

The aspA coding sequence, used as DNA guide for homologous recombination, was excised from its original vector backbone upon XbaI-SpeI double digest and it has been cloned into the pBBintФ-RFP plasmid, digested with NheI, as described in Figure 3B, and dephosphorylated.

### Chromosomal integration

The protocol described in [20] was used for site-specific recombination. Briefly, competent cells were transformed with the pInt80-649 helper plasmid [20]. Transformants were grown at 30°C and were made competent again. They were transformed with the pBBintФ integrative vector containing the part of interest as passenger and were grown on chloramphenicol plates at 30°C until colonies appeared. A single colony was propagated in LB with chloramphenicol at 37°C, 220 rpm overnight. The culture was streaked on a chloramphenicol plate and incubated overnight at 43°C. A single colony was grown in LB + chloramphenicol to yield the integrant strain. This colony was also streaked on an ampicillin plate to validate the loss of pInt80-649. Colony PCR was occasionally performed with primers P1 (CTGCTTGTGGTGGTGAAT) [15] and P2 (CTCTTACGTGCCCGATCA), which anneal upstream of the Ф80 chromosomal attB site and in the R6K origin respectively. The P1-P2 amplicon (452 bp) indicates the correct integration position, as described in [15], while negative clones show no amplicon. It is also possible to identify clones with multiple tandem copies of the integrated part via colony PCR with primers P2 (described above) and P3 (AGACGTCAGGTGGCAAAC), which anneal in opposite directions in the R6K origin and in the upstream region of the cloning site. PCR yields a 572-bp amplicon when at least two tandem copies are present in the genome, while no amplicon is produced otherwise.

If not differently stated, the chloramphenicol concentration used in all the steps of the site-specific recombination was 12.5 mg/l.

An analogous protocol was used for homologous recombination, with the exception that the passenger was present in the pBBintAsp vector and the chloramphenicol concentration used was 8 mg/l. In this case, the pInt80-649 plasmid was used to replicate the integrative plasmid via the pir-116 gene, while the Ф80 site-specific recombination machinery was not exploited, although it was expressed during the protocol.

Other integration protocols were tested (see Additional file 1: Additional integration protocols and their relative results).

### Excision of antibiotic resistance and R6K origin from an integrant strain

The protocol described in [20] was used. Briefly, the integrant strain was grown in LB and made competent. This strain was transformed with the pCP20 helper plasmid [16], plated on ampicillin plates and incubated at 30°C until colonies appeared. A single colony was propagated in non-selective LB at 37°C, 220 rpm overnight. The culture was streaked on a non-selective plate and incubated at 43°C. A single colony was propagated in non-selective LB to obtain the final single-copy integrant strain without the chloramphenicol resistance marker and the R6K origin. The loss of the antibiotic marker and the pCP20 helper were validated by streaking the obtained strain on chloramphenicol and ampicillin plates respectively. The obtained integrant strain was also assayed through colony PCR with primer pair P1 (reported above) and P4 (CTCTTACGTGCCCGATCA) for Ф80 attB targeting and with primer pair AspAFw (TGCGAGGATCGTGATGTATTTCGG) and AspARv (ATGATCTCGGGTATTCGGTCGATG) for aspA targeting. PCR products were also purified with the NucleoSpin Extract II kit and sequenced with the previously reported primer pairs or with VF2 and VR.

### Characterization of promoters

MG1655 was used as chassis for the quantitative experiments. 1 ml of M9 supplemented medium (11.28 g/L M9 salts, 1 mM thiamine hydrochloride, 2 mM MgSO4, 0.1 mM CaCl2, 0.2% casamino acids and 0.4% glycerol as carbon source) [33] was inoculated with a single colony of the desired recombinant strain, grown on a streaked LB agar plate. The culture, in a 15-ml tube, was incubated at 37°C, 220 rpm for about 20 hours and then it was 500-fold diluted in 1 ml and incubated in the same conditions as before for 6 hours. 200 μl were transferred into a 96-well microplate (Greiner), the optical density at 600 nm (OD600) was measured with an Infinite F200 microplate reader (Tecan) and the background absorbance of the medium was subtracted. The culture was diluted to an OD600 of 0.05 (pathlength of the culture volume in the microplate) in 0.5 ml and it was incubated again for 45 min. 200 μl were transferred into a 96-well microplate, incubated at 37°C for 10–15 hours in the Infinite F200 and assayed via the following kinetic cycle, programmed with the i-control™ software (Tecan): linear shaking (3 mm amplitude) for 15 s, wait for 10 s, OD600 measurement, fluorescence measurement (excitation: 535 nm; emission: 625 nm; gain: 80), sampling time: 5 min. Sterile medium and a non-fluorescent culture of MG1655 were always present in order to measure the background absorbance and fluorescence. This procedure was repeated for at least five different colonies from the freshly streaked plate for each recombinant strain.

Integrant strains were tested without antibiotic, while the plasmid-bearing strains were tested in presence of 12.5 mg/l of chloramphenicol. When required, analogous procedures were performed to test recombinant MC1061 strains.

### Data analysis for promoter activity measurement

Data were analyzed with Microsoft Excel and the MATLAB 2007b suite (MathWorks, Natick, MA). Raw absorbance and fluorescence time series were processed as previously reported [22,25,34] to obtain a value proportional to the average RFP synthesis rate per cell in exponential phase (called S_cell_).

Relative Promoter Units (RPUs) were obtained as described previously [22,25,34], by dividing the S_cell_ of the promoter of interest, in a given copy number condition, by the S_cell_ of the J23101 standard reference promoter in the same copy number condition. By definition, the RPU of the J23101 promoter is 1 in all the copy number conditions. Given a physical context, (i.e. the Ф80 locus, the aspA locus or the pSB4C5 vector), the genetic device should be present at the same copy number for all the tested promoters, as the integrated strains after marker/R6K excision have a single genomic copy of the BioBrick™ passenger in the target locus and the strains with low copy plasmid should maintain the device at the same copy number because all the plasmids are equal except the promoter to be measured. All the devices should produce the same mRNA sequence, as the predicted transcription start site is very similar for all the promoters ([34,35]) and the sequence downstream of the promoters is exactly the same (BBa_I13507). Finally, given a physical context, the growth rate of strains bearing a promoter of interest was similar to the growth rate of the strain bearing the BBa_J23101 reference promoter (data not shown). For these reasons, the RPU approach can be applied to characterize the promoters of interest of this work, as the validity hypotheses of the RPU approach are assumed to be respected.

All the coefficients of variation (CVs) were corrected for small samples by multiplying them by the *(1 + 1/(4 N))* factor, where *N* is the number of samples. Hypothesis tests were performed via MATLAB.

When assessing the statistical difference among the mean values of a group composed by more than two samples, ANOVA was performed. If the test showed a significant difference, post-hoc comparisons with the Bonferroni correction were performed via individual t-tests to compare the mean values.

In order to compute the CV within a group, the non-significantly different values were averaged and the final CV was computed on the mean values of the statistically different sub-groups.

### Evolutionary stability experiments

Two 2-ml tubes with 1 ml of M9 supplemented medium were inoculated with two single colonies from a freshly streaked LB agar plate and they were incubated as described above for 24 hours. Every 24 hours, the cultures grown to saturation were 1000-fold diluted in 1 ml of sterile medium and incubated under the same conditions as before, in order to achieve ~10 generations per day [36-38]. Every day, 1 μl of each saturated culture was also added to 200 μl of sterile medium, incubated in the Infinite F200 reader and assayed as described above. Data were analyzed as before to obtain S_cell_. The activity of a promoter over time was expressed as percentage of the activity at generation 0 (i.e. the first point of the time series).

Integrant strains were propagated and tested without antibiotic. Plasmid-bearing strains were propagated and tested in presence of 12.5 mg/l of chloramphenicol at generation 0, while they were tested with and without antibiotic for the following generations.

A freshly-inoculated non-fluorescent culture of MG1655 was always included in order to perform the fluorescence background subtraction.

After 150 generations, a glycerol stock was prepared for each evolved culture by mixing 750 μl of culture with 250 μl of 80% glycerol and stocks were long-term stored at -80°C. Evolved cultures of integrant strains and plasmid-bearing strains propagated with antibiotic were streaked on LB or LB + chloramphenicol agar plates, respectively. Plates were incubated at 37°C for about 20 hours. For each culture, 28 single colonies were picked, 1 ml of M9 supplemented medium (without antibiotic for integrant strains, with chloramphenicol for plasmid-bearing strains) was inoculated in 2-ml tubes and incubated at 37°C, 220 rpm for about 20 hours. Grown cultures were then assayed and data analyzed as described above. For each culture, 1 ml of M9 supplemented medium was also inoculated with 1 μl of glycerol stock of generation 150 and then the culture was incubated, assayed and analyzed as described above, to perform the fluorescence measurement of the whole evolved population (measured in duplicate).

Individual colonies isolated from non-evolved cultures were also assayed as a term of comparison for each recombinant strain. In this case 28 colonies were isolated by streaking the original glycerol stocks of these strains on LB agar plate.

For each culture, S_cell_ data were normalized by the median of the same recombinant strain at generation 0. Interquartile range (IQR) was used to express the variability of clones in each culture. Wilcoxon signed rank test was used to compare variability between generation 0 and 150 for all the cultures (paired samples). Wilcoxon rank sum test was used to compare variability between integrated strains and plasmid-bearing strains (unpaired samples).

## Endnotes

^1^ BioBrick™ is a trademark of The BioBricks Foundation. (http://www.biobricks.org).

## Competing interests

The authors declare that they have no competing interest.

## Authors’ contributions

SZ, LP and PM conceived the study, designed the experiments and analyzed the data. LP designed and constructed the integrative base vector. SZ generated the integrant strains. SZ performed all the promoter characterization experiments. LP and NP contributed to the evolutionary stability experiments. LP, MGCDA and PM wrote the manuscript. All authors read and approved the final manuscript.

## Supplementary Material

Additional file 1 Supplementary information. Click here for file
