# Supplementary material for: A standard vector for the chromosomal integration and characterization of BioBrick™ parts in Escherichia coli
Source: J Biol Eng. 2013 May 10;7:12. doi: 10.1186/1754-1611-7-12 (PMC3662617; doi:10.1186/1754-1611-7-12)
Supplement: Additional file 1 — Supplementary information. [file 1754-1611-7-12-S1.pdf]

SUPPLEMENTARY INFORMATION

Additional information about integrative base vector design

An early design of the pBBintΦ base vector included the default insert BBa_I52002, containing a pUC19-derived pMB1 replication origin and ccdB toxin constitutive expression cassette. It enabled the propagation of the plasmid in ccdB-tolerant strains, such as DB3.1, but not in other strains. The replication origin in this insert allowed the high-copy propagation of the plasmid in DB3.1 or similar strains even if they are not pir or pir-116.

When the default insert had to be replaced with the BioBrick™ passenger of interest, the ligation product had to be transformed into a pir or pir-116 strain because this plasmid did not contain a standard replication origin anymore. Transformants with the uncut plasmid contaminant DNA could not grow because of the ccdB toxin in BBa_I52002.

Even if the BBa_I52002 had attractive features as a default insert, we decided to use another default insert (BBa_I763007, containing an mRFP1-expression cassette driven by the PR promoter) because pBBintΦ with BBa_I52002 was not stably propagated by the DB3.1 host strain. In fact, this plasmid occasionally gave unexpected bands on agarose gel in digestion screenings, while pBBintΦ with the RFP cassette could be stably propagated by BW23474 strain, showing the correct bands every time.

Additional information about integrated BioBrick™ devices and phenotypes of recombinant strains

The pBBintΦ integrative vector was exploited to integrate a number of BioBrick™ devices in the Φ80 locus. Table S1 reports the full list of tested BioBrick™ devices, successes and failures.

In two cases, Φ80-integrant clones of MG1655 did not behave as expected, although their sequencing was correct. In particular, after integration and marker excision one clone of the J23118 promoter with RFP measurement system downstream (BBa_I13507) showed an unexpectedly higher fluorescence (>3-fold) than the other clones with the same passenger in the genome. This could be due to additional integration events in the chromosome that were not included in the P1-P4 fragment and thus could not be detected by PCR screening.

The other unexpected phenotype involved the PLlacO1 promoter (BBa_R0011) with RFP measurement system downstream (BBa_I13507). After integration and marker excision, the recombinant strain constitutively produced RFP independently of the IPTG concentration added, while the PLlacO1 was expected to show an IPTG-inducible behaviour. This could be due to lacI gene deletion that impaired the IPTG-inducible phenotype.

In both cases, the obtained phenotypes were not further studied.

Additional information about integrative vector efficiency

In this work, the pBBintΦ was also specialized to target the aspA locus, thus obtaining pBBintAsp, in the genome of *E. coli* MG1655 to construct the recombinant strains used to study the context-dependent promoter activities. The pInt80-649 helper plasmid was used to maintain the integrative plasmid after transformation via the pir-116 gene, while the Φ80 recombination machinery of the helper was not used. Although positive aspA-integrant clones could be obtained for all the BioBrick™ passengers under study, the efficiency of the integration process was significantly lower than the one measured for site-specific recombination in Φ80. In fact, the transformation plate of MG1655 (with pInt80-649 and pBBintAsp containing the BioBrick™ device of interest) incubated at 30°C showed a small set of colonies (<1%) with red phenotype, probably due to the correct RFP expression cassette in the integrative plasmid, and a large set of colonies without detectable colour. Only the red colonies could be successfully integrated in single copy, with an efficiency close to 100% in

all the required steps (i.e., helper plasmids loss, marker excision, correct phenotype and sequencing) for all the five promoters used in this work. The other colonies did not show a correct phenotype (validated fluorimetrically) and, when picked after the first incubation at 42°C on agar plate, they did not show a VF2-VR PCR product, demonstrating that the desired insert was not present in the genome. On the other hand, when pBBintAsp containing the BioBrick™ device of interest was transformed into BW23474 (a pir-116 strain) all the colonies showed the red phenotype.

Additional integration protocols and their relative results

All the integration experiments described so far and in the main text involved the protocols reported in the Methods section of the main text. However, other protocols were tested and they are described below.

- *Φ80-targeting by using pAH123 as helper plasmid.* Instead of using pInt80-649 as helper plasmid (containing a heat-inducible Φ80 phage integrase, a low copy temperature sensitive replication origin and a constitutive cassette of pir-116), we also used the pAH123 helper plasmid [15] (prepared from the CGSC#7861 strain), which is identical to pInt80-649, but lacks the pir-116 gene. In this case, the conditional-replication integrative vector could not be propagated in the host strains for integration carrying this helper.

pBBintΦ with the default insert BBa_I763007 was used in this experiment, thus targeting the BBa_I763007 device into the Φ80 attB of MG1655 and MC1061.

Competent host strains were heat-shock transformed with pAH123, propagated in selective media and made competent again. About 3 µg of miniprepped pBBintΦ-BBa_I763007 were heat-shock transformed. After heat shock, 1 ml of pre-warmed LB was added and cells were incubated at 42°C with shaking for 2 hours. Cells were plated on LB + chloramphenicol at 12.5 mg/l and incubated overnight at 42°C. Both MG1655 and MC1061 showed colonies (85 and 2000 respectively). For each strain, 3 colonies were screened and they resulted correct integrants (positive 452-bp amplicon in P1-P2 PCR) with at least 2 tandem copies of the desired part (positive 572-bp amplicon in P2-P3 PCR). All the clones lost the pAH123 helper plasmid (verified by ampicillin counterselection). The phenotype was validated fluorimetrically (data not shown), thus demonstrating that the part of interest was functional.

- *aspA-targeting in "direct suicide" mode [10].* Homologous recombination was attempted by directly transforming the non-replicating plasmid pBBintAsp (10 µg of DNA), with BBa_I763007 as passenger, into competent MG1655 and MC1061 and by plating the transformed cells on chloramphenicol (8 or 12.5 mg/l) plates. Unfortunately, no colonies could be obtained, probably because of the too low transformation efficiency of the tested strains, using heat-shock procedure (it was about 10^5 CFU per µg of DNA for MG1655 and 10^6 CFU per µg of DNA for MC1061, estimated from transformation experiments with control plasmids).

Figure S1. Colony PCR on Φ80-integrant strains with primers P1-P2 in a representative experiment. The primers anneal in opposite directions in the genome and in the integrative plasmid respectively (see Methods section in the main text). If the integration position is correct, a 452-bp amplicon is produced, while no amplicon is produced otherwise. MC = MC1061 strain; MG = MG1655 strain; G = GFP cassette as passenger (BioBrick™ device BBa_K173001); R = RFP cassette as passenger (EcoRI-PstI fragment of BBa_J23101 in the BBa_J61002 vector). Control reactions on non-recombinant (nr) strains are also reported.

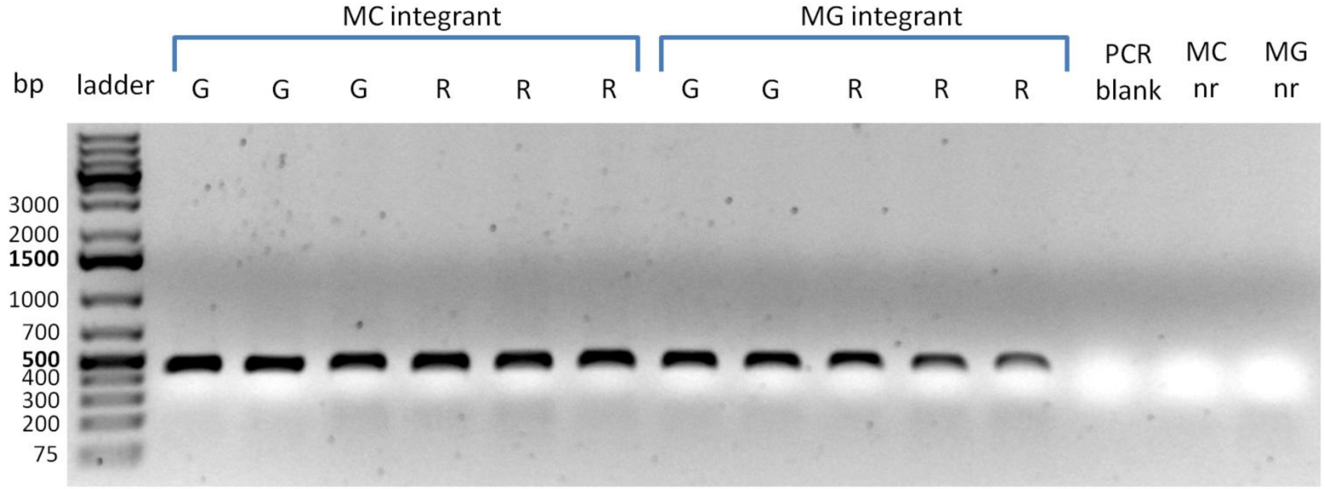

Figure S2. Colony PCR on Φ80-integrant strains with primers P2-P3 in a representative experiment. Both primers anneal in the integrative plasmid. Reaction produces a 572-bp amplicon if two or more tandem integrants are present in the genome (see Methods section in the main text). Otherwise, no amplicon is produced. MC = MC1061 strain; MG = MG1655 strain; G = GFP cassette as passenger (BioBrick™ device BBa_K173001); R = RFP cassette as passenger (EcoRI-PstI fragment of BBa_J23101 in the BBa_J61002 vector). Control reactions on non-recombinant (nr) strains are also reported.

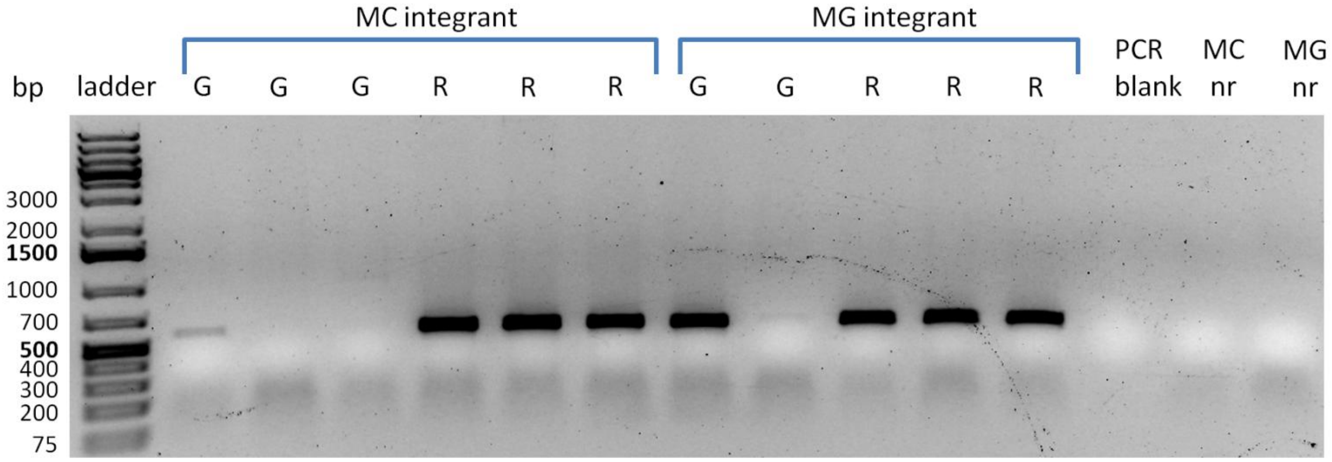

Figure S3. Colony PCR on Φ80-integrant strains, after the FRT/Flp-mediated marker excision, with primers P1-P4 in a representative experiment. Both primers anneal in the genome in opposite directions. Reaction produces a 2.3-Kbp amplicon if a single integrant of the passenger, without chloramphenicol resistance or R6K origin, is present in the Φ80 genomic locus (see Methods section in the main text). If strains do not have integrated sequences in the Φ80 genomic locus, a 546-bp amplicon is produced. MC = MC1061 strain; MG = MG1655 strain; R = RFP cassette as passenger (EcoRI-PstI fragment of BBa_J23101 in the BBa_J61002 vector). A control reaction on a non-recombinant (nr) strain is also reported.

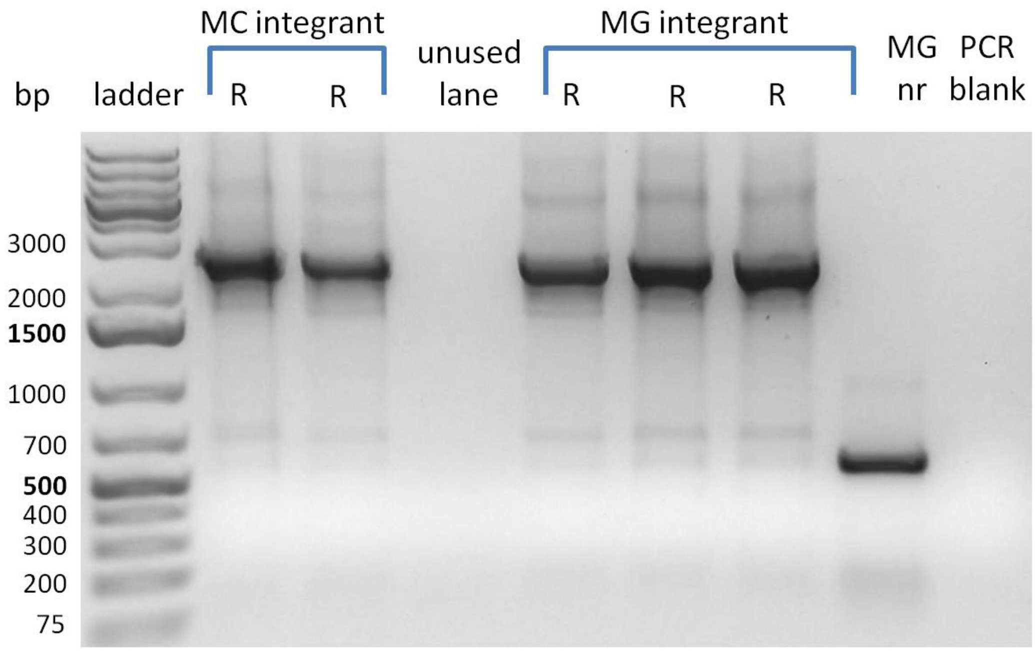

Figure S4. Evolutionary stability of the individual clones of integrant strains and low copy plasmid-bearing strains. The studied BioBrick™ promoters expressing RFP were integrated in the Φ80 and the aspA loci, propagated without antibiotic, or they were carried on the low-copy vector pSB4C5, propagated with or without antibiotic. The panels show the percent activity of all the five studied promoters in two experimental replicates (culture 1, blue line/circles and culture 2, black line/circles) over 150 generations in each condition, where 100% represents the activity measured at generation 0.

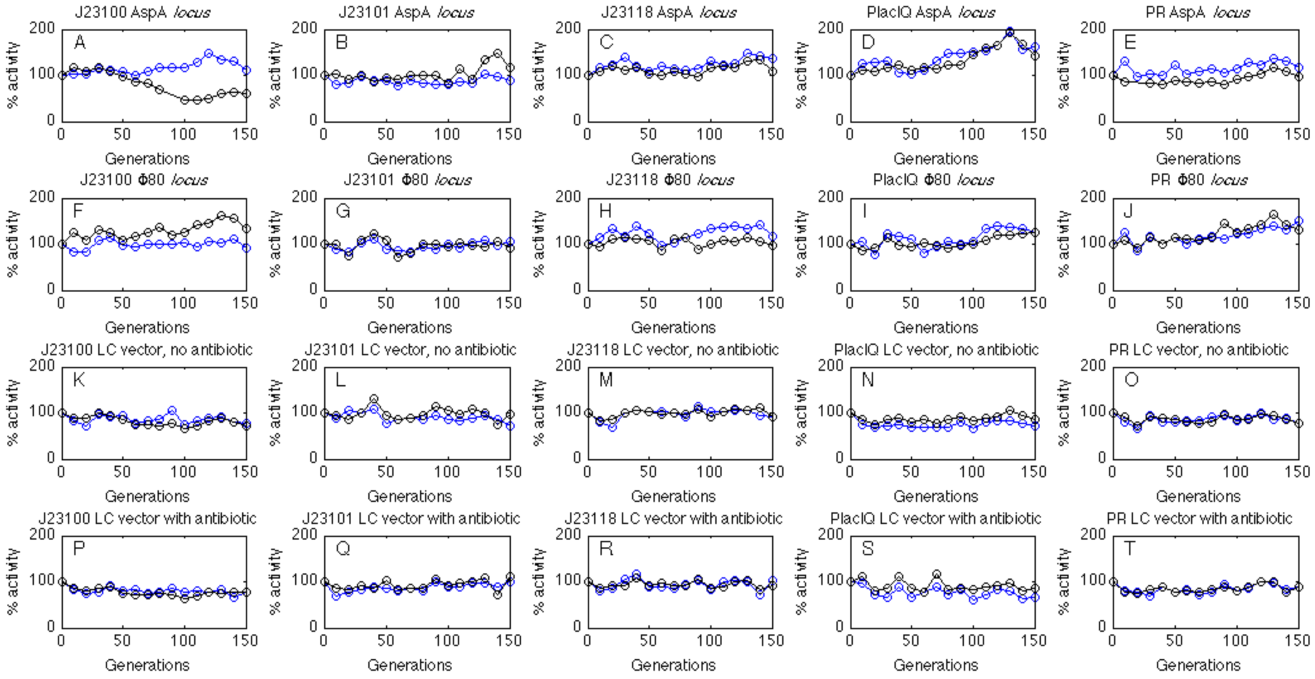

Figure S5. Dot plot showing the fluorescence variability of single clones from a non-evolved culture (generation 0, red dots) and two evolved cultures (generation 150, blue dots for culture 1; black dots for culture 2). For each investigated recombinant strain, 28 single clones isolated from glycerol stock were assayed. For each recombinant strain, data are normalized by the median fluorescence of the generation 0 culture. Circled dots indicate clones with null normalized fluorescence and the number of overlapping dots is indicated on the right of the circle.

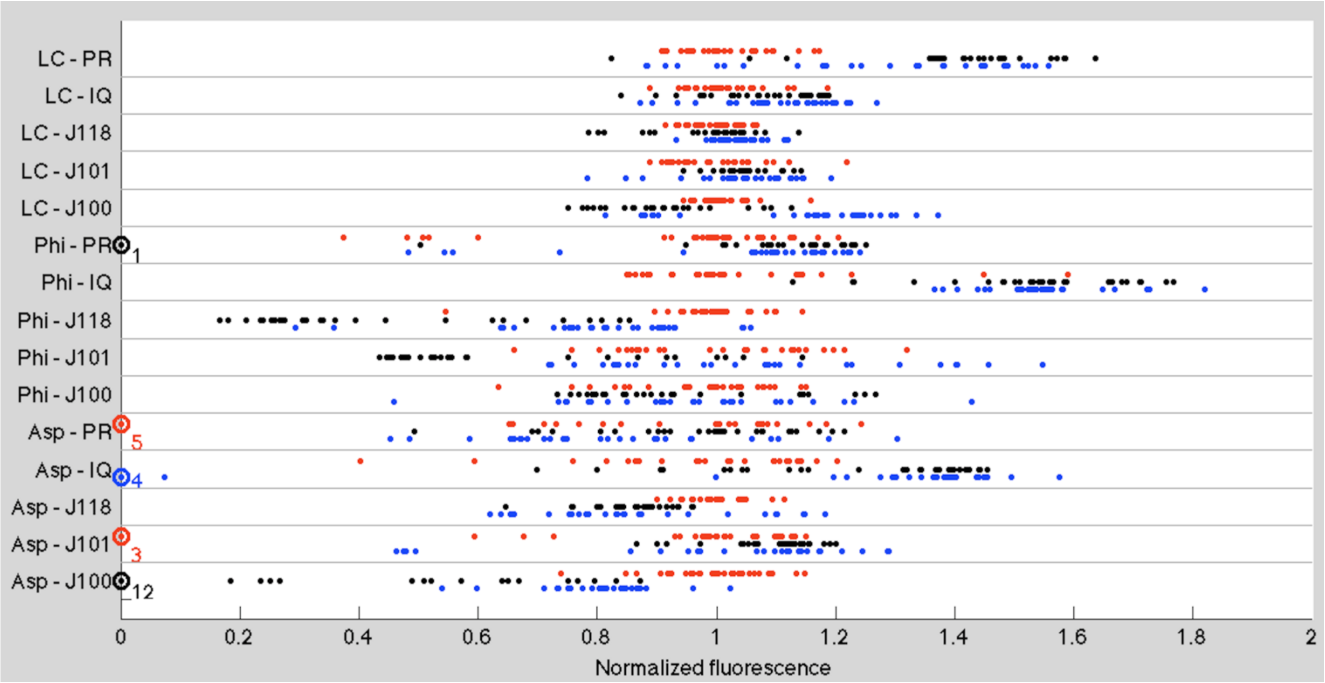

Figure S6. Comparison between the mean fluorescence of single clones from the evolved cultures and the population fluorescence of the evolved culture. Single clones were isolated from the evolved cultures (generation 150) glycerol stocks, while an aliquot (1 µl) of the same glycerol stocks was sub-cultured and used to assay the evolved cultures populations. For each evolved culture, 28 single clones were assayed. Each data point represents the mean S_{cell} of 28 clones of a specific evolved culture against the S_{cell} of the whole population (measured in duplicate) of the same culture. Solid line represents the bisector of x-y axes. S_{cell} data are shown as non-normalized values.

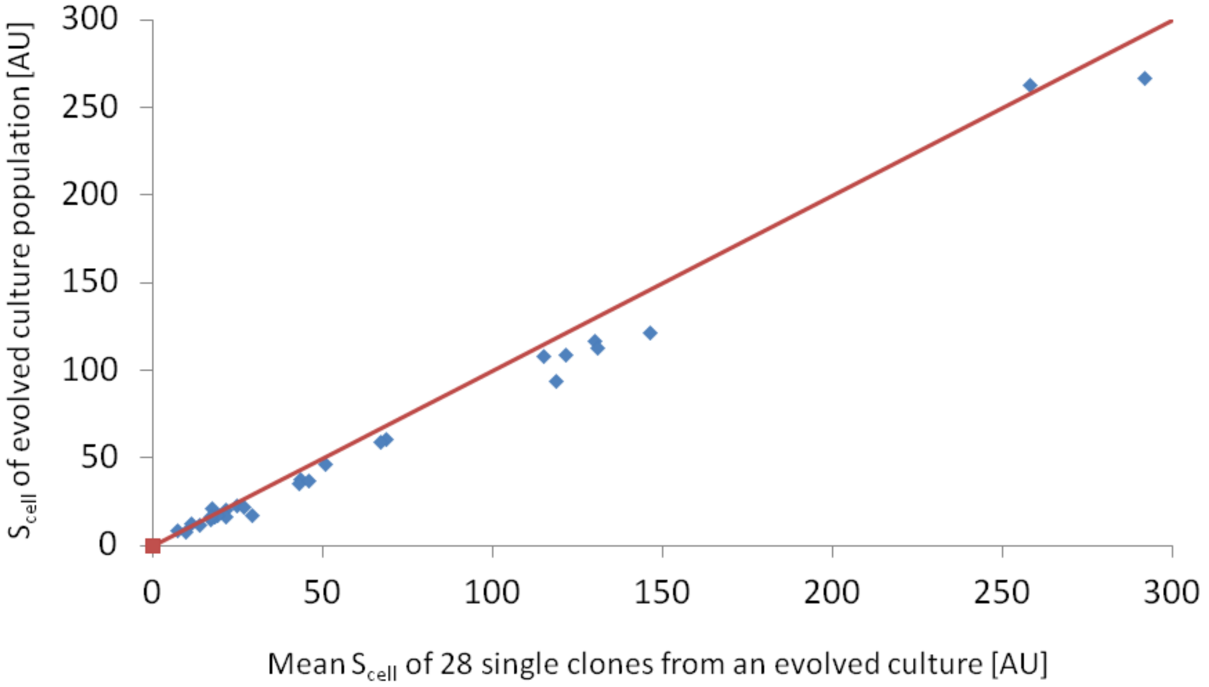

Figure S7. Assembly scheme of the integrative base vector. The chloramphenicol resistance gene, including its promoter, was assembled to a transcriptional terminator and then the resulting part was assembled to the R6K conditional replication origin. All of them were existing parts from the Registry of Standard Biologi cal Parts. The resulting CmRter-R6K sequence was digested with XbaI and SpeI, it was dephosphorylated to prevent self-ligation and it was assembled to a *de-novo* synthesized sequence (insert of pHC-attP-CS) composed by (in this order): AvrII restriction site, FRT recombination site, NheI restriction site, Φ80 attP, NheI restriction site, forward transcriptional terminator (BBa_B0053), VF2 primer binding site, reverse transcriptional terminator (BBa_B0055), EcoRI restriction site, BioBrick™ RBS BBa_B0033, PstI restriction site, forward transcriptional terminator (BBa_B0054), VR primer binding site, reverse transcriptional terminator (BBa_B0062), FRT recombination site, AvrII restriction site. AvrII, XbaI and SpeI all have compatible sticky ends. This assembly is non-directional, but the integrative vector can work with the parts ligated in both directions. The ligation orientation of the selected clone was identified by EcoRI-HindIII (Roche) digestion screening. Finally, the BioBrick™ device BBa_I763007 was ligated in place of the RBS, thus generating a standard BioBrick™ cloning site containing a default insert.

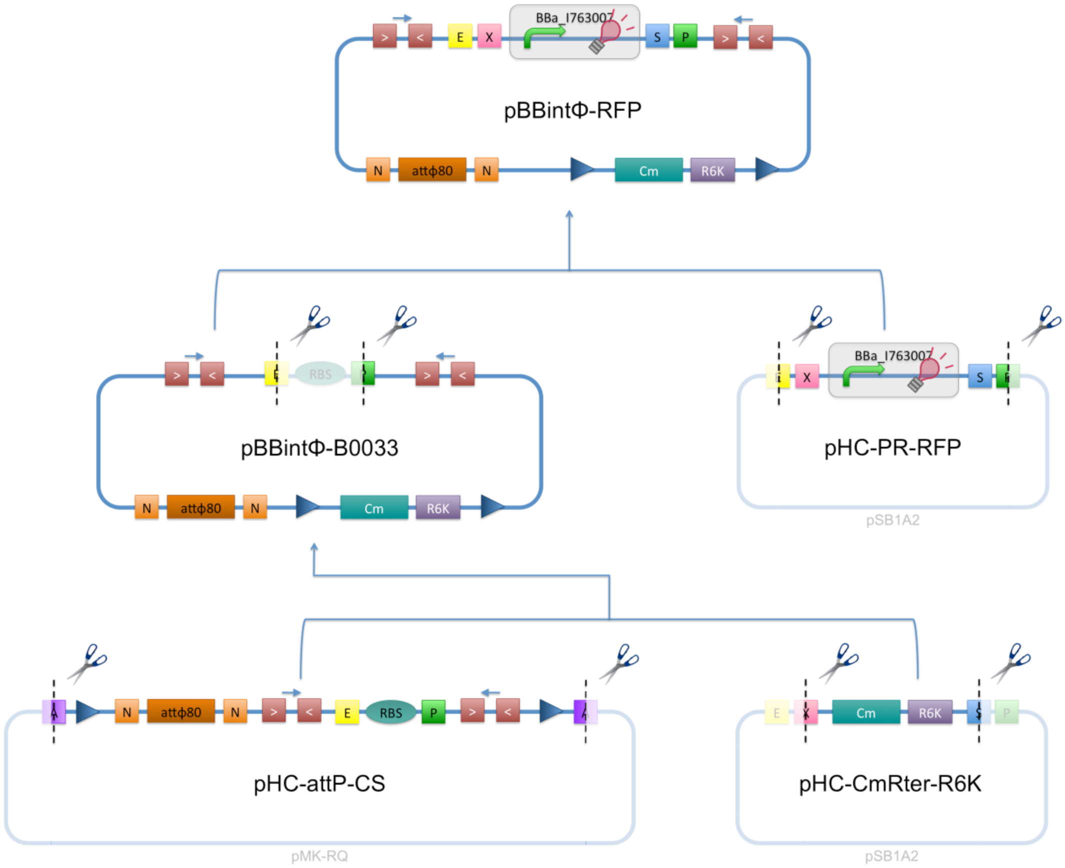

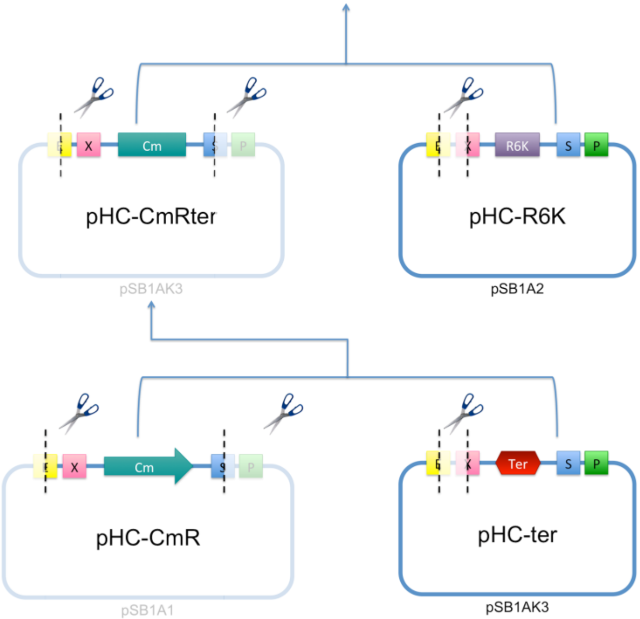

Part number	Function	Notation
BBa_G00000	BioBrick cloning site prefix	E X
BBa_G00001	BioBrick cloning site suffix	S P
BBa_I763007	PR-mRFP1 expression cassette	BBa_I763007
BBa_J72001	FRT recombination site	
BBa_B0053<br>BBa_B0054	forward transcriptional terminators	>
BBa_B0055<br>BBa_B0062	reverse transcriptional terminators	<
BBa_G00100	forward sequencing primer annealing site (VF2)	
BBa_G00102	reverse sequencing primer annealing site (VR)	
BBa_B0045	NheI restriction site	N
BBa_K300032	chloramphenicol resistance marker	Cm
BBa_J61001	R6K conditional replication origin	R6K
BBa_K300991	attϕ80 integration guide	attϕ80
BBa_B0048	AvrII restriction site	A
BBa_B0033	B0033 RBS	RBS
BBa_P1004	chloramphenicol resistance cassette without terminator	Cm
BBa_B0015	double transcriptional terminator	Ter
BBa_M31983	EcoRI restriction site	E
BBa_K300981	PstI restriction site	P

Table S1. BioBrick™ devices used as passengers in the integration experiments. If not differently stated, integration/marker excision succeeded and phenotype was correct. Passenger names are expressed as BioBrick™ codes, where the "+" sign means that the passenger is the result of a Standard Assembly of BioBrick™ parts.

Passenger	Host strain and integration locus	Notes
BBa_J107028 (BBa_J23100+BBa_I13507)	MG1655, Φ80 and MG1655, aspA	-
BBa_J107029 (BBa_J23101+BBa_I13507)	MG1655, Φ80 and MG1655, aspA	-
BBa_J107031 (BBa_J23118+BBa_I13507)	MG1655, Φ80 and MG1655, aspA	-
BBa_J107012 (BBa_I14032+BBa_I13507)	MG1655, Φ80 and MG1655, aspA	This part was also characterized in another work where the integrative vector reported here was used to construct the clones [25]
BBa_I763007 (BBa_R0051+BBa_I13507)	MG1655, Φ80; MG1655, aspA and MC1061, Φ80	-
BBa_J107010 (BBa_R0011+BBa_I13507)	MG1655, Φ80	No integrant clones with correct phenotype were obtained (see *Additional information about integrated BioBrick™ devices and phenotypes of recombinant strains*)
BBa_J107011 (BBa_I14032+BBa_E0240)	MG1655, Φ80	Phenotype could not be validated, as our instrument gave a too high background fluorescence when using the GFP filters (Excitation: 485 nm, Emission: 540 nm) on strains with a single copy of a GFP-expressing device
EcoRI-PstI fragment of BBa_J23100 in the BBa_J61002 vector	MG1655, Φ80	-
EcoRI-PstI fragment of BBa_J23101 in the BBa_J61002 vector	MG1655, Φ80 and MC1061, Φ80	This part was also characterized in another work where the integrative vector reported here was used to construct the clones [25]
EcoRI-PstI fragment of BBa_J23118 in the BBa_J61002 vector	MG1655, Φ80	-
BBa_K173001 (BBa_J23101+BBa_E0240)	MG1655, Φ80 and MC1061, Φ80	Phenotype could not be validated, as our instrument gave a too high background fluorescence when using the GFP filters (Excitation: 485 nm, Emission: 540 nm) on strains with a single copy of a GFP-expressing device
EcoRI-PstI fragment of BBa_F2620 in the BBa_J61002 vector	MG1655, Φ80	This part was also characterized in another work where the integrative vector reported here was used to construct the clones [25]

Table S2. Interquartile ranges (IQRs) of the data represented in Figure S6. For each culture, IQR is computed on normalized fluorescence data of 28 single clones, isolated from glycerol stock of cultures at generation 0 and generation 150. Asp, Phi and LC indicate the aspA integration locus, the Φ80 integration locus and the pSB4C5 low copy vector contexts, respectively. For generation 150 cultures, 1 and 2 indicate culture 1 and 2 respectively.

**Culture (generation 150)**	**IQR [normalized fluorescence]**
Asp - J100-1	0.08413811
Asp - J100-2	0.64598618
Asp - J101-1	0.18707548
Asp - J101-2	0.0857181
Asp - J118-1	0.24213815
Asp - J118-2	0.06023159
Asp - IQ-1	0.19160027
Asp - IQ-2	0.35056591
Asp - PR-1	0.25722746
Asp - PR-2	0.18234194
Phi - J100-1	0.23613991
Phi - J100-2	0.21115181
Phi - J101-1	0.38690393
Phi - J101-2	0.36924286
Phi - J118-1	0.16111558
Phi - J118-2	0.37701949
Phi - IQ-1	0.09833782
Phi - IQ-2	0.18256188
Phi - PR-1	0.10716522
Phi - PR-2	0.13581053
LC - J100-1	0.32338727
LC - J100-2	0.11037756
LC - J101-1	0.10225783
LC - J101-2	0.04550428
LC - J118-1	0.06266528
LC - J118-2	0.07503204
LC - IQ-1	0.12393891
LC - IQ-2	0.12748135
LC - PR-1	0.42055468
LC - PR-2	0.12308644
**Culture (generation 0)**	**IQR [normalized fluorescence]**
Asp - J100	0.11236565
Asp - J101	0.16545605
Asp - J118	0.06615725
Asp - IQ	0.25396722
Asp - PR	0.38741945
Phi - J100	0.1563073
Phi - J101	0.26614573
Phi - J118	0.05069474
Phi - IQ	0.23357529
Phi - PR	0.1284302
LC - J100	0.03683607
LC - J101	0.11510437
LC - J118	0.05672519
LC - IQ	0.08143892
LC - PR	0.1035448
